# Results from the Pan-European CIEMAR study: Local Tumour Control After Microwave Ablation of Colorectal Liver Metastases and Exploratory Associations with Ablation Margins and Confirmation Software

**DOI:** 10.1007/s00270-026-04578-1

**Published:** 2026-09-06

**Authors:** Martijn Meijerink, Hannah Schulz, Asmund A. Fretland, Reto Bale, Dirk Arnold, Tugsan Balli, Carlo Gazzera, Stefan Staettner, Thomas Helmberger, Thomas Vogl, Anna Kafloula, Bleranda Zeka, Maria Urdaniz, Franco Orsi, Philippe L. Pereira

**Affiliations:** 1 Department of Radiology and Nuclear Medicine, Amsterdam University Medical Centers, Location VUmc De Boelelaan 1117, 1081 HV Amsterdam, The Netherlands; 2 The Intervention Centre, and Department of HPB surgery, Oslo University Hospital, Oslo, Norway; 3 Institute of Clinical Medicine, University of Oslo, Oslo, Norway; 4 Department of Radiology, Section of Interventional Oncology-Microinvasive Therapy (SIP), Medical University of Innsbruck, Anich Str. 35, 6020 Innsbruck, Austria; 5 Oncology and Hematology, Asklepios Tumorzentrum Hamburg, AK Altona, Paul-Ehrlich-Str. 1, 22763 Hamburg, Germany; 6 Department of Radiology, Çukurova University School of Medicine, Saricam, 01330 Adana, Turkey; 7 Department of Diagnostic and Interventional Radiology, A.O.U. Città Della Salute E Della Scienza Di Torino, Corso Bramante 88, 10126 Turin, Italy; 8 Department of General, Visceral and Vascular Surgery, SKG Kliniken Voecklabruck and Gmunden, Gmunden, Austria; 9 Department of Radiology Neuroradiology and Minimal-Invasive Therapy, Klinikum Bogenhausen, Englschalkinger Str. 77, 81925 Munich, Germany; 10 Department of Radiology, University Hospital Frankfurt, Frankfurt, Germany; 11 Next Research GmbH and Cardiovascular and Interventional Radiological Society of Europe, Neutorgasse 9, 1010 Vienna, Austria; 12 Divisione Di Radiologia Interventistica, Istituto Europeo Di Oncologia, Istituto Di Ricovero E Cura a Carattere Scientifico (IRCCS), Milan, Italy; 13 Center of Radiology, Minimally Invasive Therapies and Nuclear Medicine, SLK-Kliniken GmbH, Heilbronn, Germany

**Keywords:** Microwave ablation, Colorectal liver metastases, Metastatic colorectal cancer, Confirmation software, Safety margin

## Abstract

**Purpose:**

The CIRSE Emprint Microwave Ablation (MWA) Registry (CIEMAR) was designed to evaluate real-world effectiveness of MWA for colorectal liver metastases (CRLM). This analysis reports on local tumour control and includes post-hoc analyses of ablation margin size and confirmation software use.

**Materials and Methods:**

Patients were eligible if MWA was indicated as part of a curative-intent strategy for ≤9 local treatment-naïve CRLM measuring ≤3 cm, with no extrahepatic disease except ≤5 lung nodules. Baseline, treatment and follow-up data from September 2019 to December 2025 were analysed. Ablation margin categories were reported locally according to centre-specific practice. The primary endpoint was the 1-year local tumour control rate after the last MWA.

**Results:**

In total, 491 patients with 1083 CRLM from 30 sites in 11 countries were included. Reported margins were <5mm (12.3%), 5-10mm (35.5%) or >10mm (44.5%); for 1.5% and 6.2% of lesions, ablation was incomplete and data were missing, respectively. The 1-year local tumour control rate was 88.9% (95% CI 83.2–94.6) on a per-lesion basis and 81.2% (95% CI 77.2–84.6) on a per-patient basis. Larger margins were independently associated with lower odds of local failure (5–10 mm: OR 0.30; >10 mm: OR 0.10; both *p*<0.01 vs <5 mm). Use of confirmation software was associated with wider reported margins (*p*<0.001).

**Conclusion:**

MWA for CRLM achieved high 12 month local tumour control. These findings support routine verification that adequate ablation margins have been achieved, while exploratory association between confirmation software use and wider margins warrants further confirmation.

*Trials registration*: NCT03775980.

**Graphical Abstract:**

**Figure:**
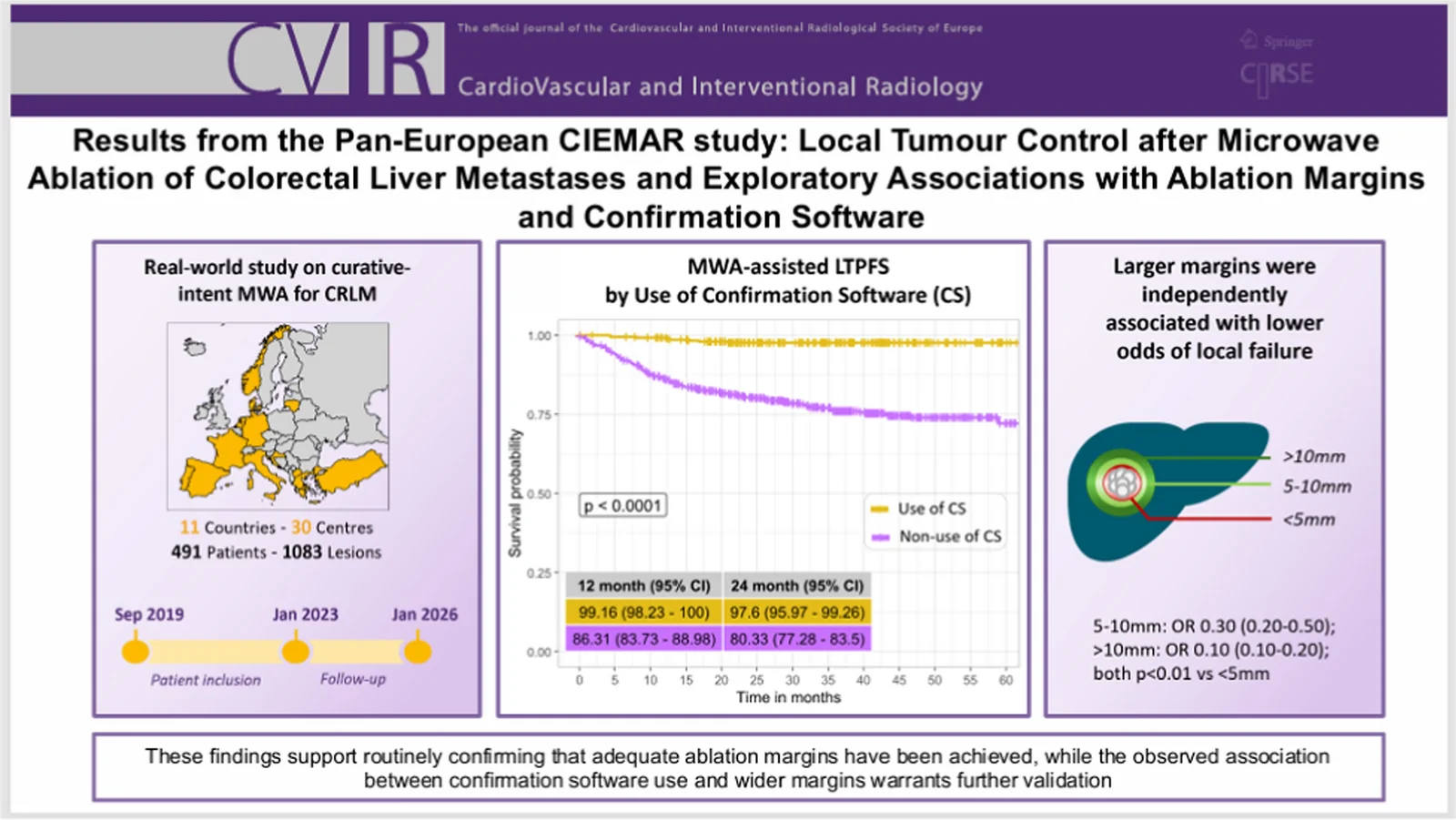

**Supplementary Information:**

The online version contains supplementary material available at https://doi.org/10.1007/s00270-026-04578-1.

## Introduction

While surgical resection of liver metastases has long been regarded as the standard of care for resectable colorectal cancer liver metastases (CRLM), image-guided thermal ablation is now considered to provide comparable oncological outcomes for small lesions (≤ 3 cm) when all visible disease can be ablated with adequate ablation margins, while offering a less invasive, safer, and more cost-effective treatment option for selected patients [1–3]. Among ablative modalities, microwave ablation (MWA) has gained prominence as an alternative to resection due to its ability to achieve large, predictable and reproducible ablation margins and its favourable safety profile [4–8].

Achieving durable local tumour control (LTC) is recognised as a key determinant of long-term treatment success. Across thermal ablation modalities, available evidence indicates that 5–10 mm ablation margins are a key technical factor influencing local outcomes [9–11]. However, reliable intra-procedural margin assessment remains challenging when based solely on visual comparison of pre- and post-ablation imaging, and technical failure of the ablation zone is often a major driver of local recurrence [12–14].

To address this limitation, dedicated confirmation software platforms have been introduced to facilitate intraprocedural image registration and quantitative assessment of the ablation zone [9, 12, 13]. These tools allow operators to verify lesion coverage and margin adequacy during the procedure, potentially enabling immediate re-ablation when necessary and reducing the risk of undertreatment. Considering this, margin-verification technology may have a substantial impact on LTC [14], yet real-world evidence on its effectiveness remains limited.

The aim of this multicentre Europe-wide study was to evaluate LTC in everyday clinical practice. LTC was evaluated by assessing the tumour control rate after the last MWA, MWA-assisted local tumour progression-free survival (LTPFS), and the rate of lesions assessed as untreatable by thermal ablation (time to untreatable progression by ablation, TTUP).

## Methods

### Study Design

The multicentre, prospective, single-arm observational study, CIEMAR, included patients from 30 centres in 11 countries between September 2019 and January 2023. The study was prospectively designed to understand the real-world effectiveness of MWA using Emprint™ or Emprint HP™ as the standard of care for CRLM treatment with curative intent. All other technical aspects of the treatment were done according to the treating physician’s discretion.

The CIEMAR methodology detailing centre and patient inclusion criteria, as well as study timelines, study endpoints, and the planned statistical analysis were published in 2024 [15].

The study was conducted in accordance with the principles of the Declaration of Helsinki, as well as applicable national and international regulations. All participating centres received ethics committee approval, and all patients were required to sign an informed consent form.

### Patient Inclusion

Eligible patients were ≥18 years, diagnosed with CRLM, and had been referred to curative-intent MWA by a multidisciplinary tumour board. A maximum of 9 locally treatment-naïve lesions with a maximum diameter of 3 cm could be enrolled in the study. An intention to treat all other visible disease within 8 weeks was required. Exclusion criteria were life expectancy of less than 6 months, advanced liver disease or evidence of liver insufficiency, pregnancy, the presence of extrahepatic disease except for up to 5 lung nodules, ongoing infection or active cancers other than the primary colorectal cancer, receiving simultaneous bowel surgery, irreversible electroporation, RFA (radiofrequency ablation), SBRT (stereotactic body radiotherapy), cryoablation, high-intensity focused ultrasound, or other local treatment except resection. The full criteria were published in 2024 [15].

### Assessments

The primary endpoint was defined as the fixed landmark 12 month local tumour control rate per target CRLM after the last MWA treatment. For this, the proportion of target CRLM without local failure at 1 year ± 45 days after the last MWA treatment was used. Secondary endpoints included MWA-assisted LTPFS, defined as the time from the first MWA to the time of local failure of a target lesion, which was defined as residual disease or recurrence that could not be successfully retreated with MWA, and TTUP. Assessments were done locally according to the investigators’ routine protocols including determining treatment completion and technical success.

Procedural volume was defined as the number of MWA treatment sessions performed per site during the study period.

### Data Collection

Data were collected using OpenClinica 4 (OpenClinica, LLC, Needham, MA) via electronic case report forms. Details on the timeline of data collection were published in 2024 [15]. Briefly, baseline data were collected before the first MWA treatment session (median: 15 days, interquartile range: 3–31 days). Data on technical treatment success, guidance methods, and site-reported ablative margins per target lesion were collected. For procedures using confirmation software, the date of margin assessment was generally the treatment date. For procedures without confirmation software, the method and timing of the initial margins assessment were not captured, however the date of confirmation of complete ablation (median interval from treatment: 30 days; interquartile range: 4–41 days) was collected.

To ensure data quality, automated data checks and verifications were used together with quarterly remote monitoring calls to resolve inconsistencies. No source data validation was carried out. No imputation for missing data was done.

### Statistical Methods

The sample size was determined to estimate the primary endpoint with the prespecified precision; all secondary and additional analyses should therefore be considered exploratory. For the primary endpoint, local tumour control rates were calculated among evaluable lesions, and the corresponding 95% confidence interval was estimated using the Wilson score method. Loss of local tumour control within 12 months was defined at the lesion level as recurrence or residual disease occurring within 410 days after the last MWA treatment. Lesions were considered controlled if a “complete ablation” assessment was documented between 320 and 410 days post-treatment or later. Lesions with residual disease recorded before day 320 and without subsequent re-ablation were classified as having lost local tumour control. Lesions without recurrence or residual disease but with no follow-up assessment later than 320 days after the last treatment, lesions from patients lost to follow-up, or from patients who died without evidence of local recurrence were considered dropouts. For the patient-level analysis, patients were classified as having maintained local tumour control only if all evaluable treated lesions remained locally controlled.

Deterministic sensitivity analyses were performed at the lesion level. Non-evaluable lesions were alternatively classified as controlled (best-case assumptions), classified as failures (worst-case assumptions), or, in a conservative death-related scenario, classified as failures only when the patient died from disease progression within 12 months.

To identify factors associated with loss of local tumour control, a logistic regression analysis was performed. Baseline and treatment variables with a univariable *p*-value <0.10 were included in the initial multivariable model. A backward selection procedure based on both *p*-value and Akaike Information Criterion (AIC) was then applied to derive the final multivariable model. For this analysis, imaging guidance was collapsed into three categories to ensure adequate sample sizes and stable model estimates: (1) CT or MRI guidance, with or without additional ultrasound or other adjunctive guidance techniques, including fusion guidance where US was used in an intraprocedural workflow based on CT/MRI acquired during the same treatment session; (2) intraoperative or laparoscopic ultrasound guidance, including open palpation; and (3) percutaneous ultrasound guidance.

In an exploratory centre-level analysis, the 12 month local tumour control rate was further described according to procedural volume and use of confirmation software. Sites were grouped into three procedural-volume categories: very low volume (< 5 treatment sessions), low volume (5–30 treatment sessions), and high volume (> 30 treatment sessions). A threshold of >30 treatment sessions was considered clinically relevant for distinguishing sites with greater procedural experience and resulted in groups with meaningful sample sizes. The lower cut-off of five treatment sessions was used to distinguish sites with very limited exposure, where centre-level outcome estimates are expected to be unstable and strongly influenced by single events. Centres were further classified according to use of confirmation software, defined as use of software-based margin confirmation in more than one treated lesion at the respective centre.

Time-to-event outcomes were analysed using the Kaplan–Meier method, and results were reported as the 12- and 24 month estimates with corresponding 95% confidence intervals (CIs). TTUP by ablation was analysed as described in 2024 [15]. MWA-assisted LTPFS was added to the analysis plan and defined as the time from the first MWA ablation of a lesion until local recurrence or residual disease at the lesion level that was not successfully retreated with MWA. Lesions without an event until study exit or death were censored at the date of the last contact and lesions that were resected but were considered technically treatable by ablation were censored at the date of the respective follow-up. Kaplan–Meier curves were generated for MWA-assisted LTPFS and TTUP in the full cohort and according to confirmation software use. In addition, MWA-assisted LTPFS was further explored in lesion-size subgroups (< 10 mm, 10–20 mm, and >20 mm), both in the full cohort and stratified by confirmation software use. MWA-assisted LTPFS and TTUP were compared between lesions treated with and without confirmation software using the log-rank test. The relationship between confirmation software use (yes/no) and the site-reported ablation margin category (< 5 mm, 5–10 mm, >10 mm) was analysed using the Chi-square test.

All statistical analyses were carried out by B. Z. (neuroscientist by formal training with additional certifications for biostatistics) using R Studio version 2025.05.1 (Posit, 2009, Boston) under R 4.5.1 (R Core Team, 1993; R Foundation for Statistical Computing, 2003, Vienna, Austria) and a two-sided *p*-value <0.05 was considered statistically significant.

## Results

### Demographics, Treatment Characteristics and Safety

Data were collected from 30 centres across 11 European countries. A total of 500 patients referred for curative-intent CRLM treatment with MWA using Emprint TM or Emprint HP TM were enrolled (intent-to-treat population). Nine patients did not receive the MWA treatment as planned, amounting to a modified intent-to-treat population of 491 patients who received treatment for 1083 lesions.

The median age was 66 years (range: 29–92) and 64.6% of patients were male. 91% of the patients had ECOG 0–1. The primary tumour was right-sided in 26.1% of the patients. Roughly half (47.3%) of the patients had 1 target lesion. 67.4% of target lesions were <20 mm, and 19.2% of target lesions were considered close to a critical structure. Guidance method at baseline was conventional cross-sectional imaging (CT or MRI), with or without complementary non-fused US in 588 lesions (54.3%), percutaneous US guidance without cross-sectional imaging in 192 (17.7%), intraoperative/laparoscopic US guidance without cross-sectional imaging in 130 (12.0%), real-time US–CT/MRI fusion guidance in 113 (10.4%), stereotactic navigation- or robot-assisted guidance in 53 (4.9%), or open palpation alone in 7 (0.6%). A site-reported safety margin of >10 mm was recorded for 44.5%, 5–10 mm for 35.5%, and <5 mm for 12.3% of lesions. Confirmation software was used for 34.1% of lesions. Detailed patient and lesion characteristics, as well as treatment information are provided in Table 1, Table 2 and Supplementary Table 1.

**Table 1:** Baseline characteristics

Participant characteristics	n (%) or value
**Participants**	
Total	491
**Age (years)**	
Mean (SD)	64.96 (11.68)
Median (IQR)	66 (57—74)
Min	29
Max	92
**Sex**	
Male	317 (64.6)
Female	174 (35.4)
**ECOG performance status**	
0	347 (70.7)
1	99 (20.2)
2–5	14 (2.8)
Not available	31 (6.3)
**Lung metastases**	
Yes	58 (11.8)
No	433 (88.2)
**Tumour Biology (284 – 57.8% available)**	
Double mutation	16 (5.6)
Ras mutation	120 (42.3)
BRAF mutation	9 (3.2)
Double wild type	139 (48.9)
**CEA value (categorised)**	
≤ 200 ng/ml	414 (84.3)
> 200 ng/ml	3 (0.6)
Not available	74 (15.1)
**Primary tumour location**	
Right Colon—Before the splenic flexure	128 (26.1)
Left Colon—After the splenic flexure	194 (39.5)
Rectum—From the anal verge to 15 cm above	169 (34.4)
**Diagnosis of liver metastases**^**1**^	
Synchronous	198 (40.3)
Metachronous	293 (59.7)
**Prior systemic therapy after the diagnosis of liver metastases**	
Yes	254 (51.7)

Abbreviations; SD: standard deviation, IQR: interquartile range, ECOG: eastern cooperative oncology group, CEA: carcinoembryonic antigen

^1^Diagnosis is synchronous when the liver metastases were diagnosed before, or until 7 days after the primary tumour date, metachronous otherwise

**Table 2:** Lesion and treatment characteristics

Lesion and treatment characteristics		n (%) or value
**Lesions Treated**		
Total		1083
**Number of lesions treated per patient**		
1		232 (47.3)
2		116 (23.6)
≥3		143 (29.1)
**Number of treatments per lesion**		
One initial treatment		1059 (97.8)
Two initial treatments		24 (2.2)
One retreatment during follow-up		30 (2.8)
Two retreatments during follow-up		2 (0.2)
**Location**		
Segment I		14 (1.3)
Segment II		97 (9.0)
Segment III		69 (6.4)
Segment IVa		128 (11.8)
Segment IVb		33 (3.0)
Segment V		120 (11.1)
Segment VI		166 (15.3)
Segment VII		208 (19.2)
Segment VIII		246 (22.7)
Not evaluable micronodules		2 (0.2)
**Size (categorised)**		
<20 mm		730 (67.4)
20—30 mm^1^		350 (32.3)
Not evaluable micronodules		3 (0.3)
**Lesion close to critical structure**		
No		875 (80.8)
Yes^2^		208 (19.2)
**Maximum ablation power in Watt (categorised)**		
<50 Watt		4 (0.4)
50–100 Watt		828 (76.5)
101–150 Watt		238 (22.0)
Missing		13 (1.2)
**Safety margin around the ablation zone**		
**Ablation not complete**^**3**^		16 (1.5)
<5 mm		133 (12.3)
5—10 mm		385 (35.5)
>10 mm		482 (44.5)
Missing		67 (6.2)
**Confirmation software used**		
No		693 (64)
Yes		369 (34.1)
Missing		21 (1.9)
**Ablation treatment method**		
Percutaneous		929 (85.8)
Surgically (open)		106 (9.8)
Laparoscopic		48 (4.4)
**Image guidance method**		
Conventional cross-sectional imaging (CT or MRI), with or without complementary non-fused US^4^		588 (54.3)
Real-time US–CT/MRI fusion guidance^5^		113 (10.4)
Intraoperative/laparoscopic US guidance without cross-sectional imaging		130 (12.0)
Percutaneous US guidance without cross-sectional imaging		192 (17.7)
Stereotactic navigation- or robot-assisted guidance		53 (4.9)
Open palpation alone		7 (0.6)

^1^All lesions except two were maximal 30 mm long according to the inclusion criteria. One lesion was 36 mm which was treated surgically and thus could exceed this limitation and one lesion was maximal 30 mm at baseline but increased to 50 mm in the time between baseline and MWA treatment

^2^≤3 mm to large vessels, ≤5 mm to the main bile duct, ≤10 mm to bowel, stomach, adrenal glands, diaphragm, heart, or gallbladder

^3^Reasons for incomplete ablation: Adjacent blood vessels: 5, Adjacent critical structures: 3, Difficulty getting optimal antenna placement: 2, Difficulty visualising target lesion: 4, Unclear: 2

^4^118 used US for probe-placing; 6 used CT and surgery, 5 MRI

^5^fusion guidance refers to an intraprocedural workflow based on CT/MRI acquired during the same treatment session

Abbreviations; CT: computer tomography, MRI: magnetic resonance image, US: ultrasound

Procedure-related or likely procedure-related AEs were reported in 80 participants (16.3%), while serious procedure-related or likely procedure-related events (grade 3–4) were observed in 26 participants (5.3%). No related grade 5 AEs were reported (Supplementary Table 2).

### 1-year Local Tumour Control Rate and Associated Factors

Looking at the 1-year local control rate after the last MWA, 947 of 1083 treated lesions (87.4%) had reached evaluable follow-up, while 136 lesions (12.6%) were considered dropouts due to incomplete follow-up (38; 3.5% of all lesions) or competing risk due to death (50; 4.6% of all lesions) before 12 months (Table 3). For 40 (3.7%) non-evaluable lesions due to death before 12 months, “death due to disease progression” was given, however whether the target lesion(s) had progressed could not be evaluated.

**Table 3:** Local tumour control rate and predictor analysis

Variable	N (%)	95% CI		
Per lesion^*^				
Treated Lesions	1083			
12 month Follow-up	947			
Lesions controlled	842 (88.9)	83.2—94.6		
Per patient				
Treated Patients	491			
12-month Follow-up	430			
Patients with all lesions controlled	349 (81.2)	77.2–84.6		
Variable	Controlled	Not controlled	OR (univariable)	OR (multivariable)
ECOG status				
0	600 (76.8)	69 (67.6)	–	–
≥1	181 (23.2)	33 (32.4)	1.6 (1.0–2.5, *p*=0.04)	2.5 (1.5–4.3, *p*<0.01)
Guidance method^1^				
CT or MRI ± US or other	591 (70.2)	55 (52.4)	–	–
US or open palpation (laparoscopic/surgery)	115 (13.7)	7 (6.7)	0.7 (0.3–1.4, *p*=0.31)	0.9 (0.3–2.0, *p*=0.75)
US (percutaneous)	134 (15.9)	43 (41.0)	3.5 (2.2–5.4, *p*<0.01)	3.6 (2.2–5.9, *p*<0.01)
Lesion Size				
≤20 mm	654 (77.8)	59 (56.2)	–	–
>20 mm	187 (22.2)	46 (43.8)	2.7 (1.8–4.1, *p*<0.01)	1.7 (1.0–2.7, *p*=0.03)
Margins Size				
<5 mm	84 (10.5)	42 (41.2)	–	–
5–10 mm	307 (38.5)	41 (40.2)	0.3 (0.2–0.4, *p*<0.01)	0.3 (0.2–0.5, *p*<0.01)
>10 mm	406 (50.9)	19 (18.6)	0.1 (0.1–0.2, *p*<0.01)	0.1 (0.1–0.2, *p*<0.01)
Age (categorised)				
<70	562 (66.7)	59 (56.2)	–	–
≥70	280 (33.3)	46 (43.8)	1.6 (1.0–2.4, *p*=0.03)	–
Lesion next to critical structure				
No	691 (82.1)	73 (69.5)	–	–
Yes	151 (17.9)	32 (30.5)	2.0 (1.3–3.1, *p*<0.01)	–
Maximal ablation power				
<50 Watt	4 (0.5)	0 (0.0)	–	–
50–100 Watt	645 (77.7)	83 (79.0)	272,572.0 (0.0-NA, *p*=0.98)	–
101–150 Watt	181 (21.8)	22 (21.0)	257,458.4 (0.0-NA, *p*=0.98)	–
Ablation time				
1–5 min	211 (27.8)	25 (23.8)	–	–
6–9 min	286 (37.7)	48 (45.7)	1.4 (0.9–2.4, *p*=0.19)	–
10–14 min	172 (22.7)	20 (19.0)	1.0 (0.5–1.8, *p*=0.95)	–
≥15 min	90 (11.9)	12 (11.4)	1.1 (0.5–2.3, *p*=0.75)	–
Liver Metastases Diagnosis				
Metachronous	461 (54.8)	65 (61.9)	–	–
Synchronous	381 (45.2)	40 (38.1)	0.7 (0.5–1.1, *p*=0.17)	–
Primary Tumour Location				
Left	350 (41.6)	34 (32.4)	–	–
Right	210 (24.9)	31 (29.5)	1.5 (0.9–2.5, *p*=0.11)	–
Rectum	282 (33.5)	40 (38.1)	1.5 (0.9–2.4, *p*=0.12)	–
Sex				
Female	290 (34.4)	31 (29.5)	–	–
Male	552 (65.6)	74 (70.5)	1.3 (0.8–2.0, *p*=0.32)	–

Number in dataframe = 947, Number in model = 841, Missing = 106, AIC = 517.2, C-statistic = 0.799, H&L = Chi-sq(8) 8.34 (*p*=0.401)

^*^136 (12.6%) drop-out lesions:

from patients lost to follow-up: 63 (5.8%), of those 38 (3.5%) <12 mo after treatment

from patients who died: 73 (6.7%), of those 50 (4.6%) < 12 mo after treatment

^1^For the regression analysis, conventional CT/MRI guidance and CT/MRI-based multimodality guidance were combined because of the limited number of fusion-guided procedures

Abbreviations; OR: odds ratio, CI: confidence interval, ECOG: eastern cooperative oncology group, CT: computer tomography, MRI: magnetic resonance image, US: ultrasound

Among evaluable lesions, the local tumour control rate at 1 year was achieved in 842 lesions (88.9%; 95% CI 83.2–94.6). The per-patient local tumour control rate was 81.2% (95% CI: 77.2%–84.6%; Table 3).

A deterministic sensitivity analysis for the 136 non-evaluable lesions yielded estimated 12 month local control rates of 90.3% under the best-case assumption, 77.7% under the worst-case assumption, and 85.3% when the 40 lesions from patients who died due to disease progression were classified as failures (Supplementary Table 3).

In the multivariable logistic regression model, ECOG status, guidance method, target lesion size, and ablation margin size remained independently associated with the 1-year local tumour control rate. Regarding guidance method, percutaneous US-only guidance was associated with higher odds of local failure compared with cross-sectional imaging-based or multimodality guidance including CT/MRI fusion procedures (OR 3.6, 95% CI 2.2–5.9, *p*<0.01), while procedures performed using ultrasound (laparoscopic/surgical) or open palpation alone did not have higher odds of local failure. Lesions from patients with ECOG ≥1 and lesions >20 mm also had higher odds of local failure (OR 2.5, 95% CI 1.5–4.3, *p*<0.01, and OR 1.7, 95% CI 1.0–2.7, *p*=0.03).

In contrast, compared with ablation margins < 5 mm, margins of 5–10 mm and > 10 mm were associated with substantially lower odds of local failure (OR 0.3, 95% CI 0.2–0.5, *p*<0.01, and OR 0.1, 95% CI 0.1–0.2, *p*< 0.01, respectively).

In a supplementary analysis using a margin of 5–10 mm as the reference category, margins >10 mm were associated with a further reduction in the odds of local failure (OR 0.3, 95% CI 0.2–0.6, *p*<0.01), while margins <5 mm were associated with increased odds of local failure (OR 3.2, 95% CI 1.9–5.5, *p*<0.01; Supplementary Table 4).

### Confirmation Software and Guidance Methods

The use of confirmation software was strongly associated with site-reported margin category (*p*< 0.001, Table 4). Among lesions treated with confirmation software, a margin >10 mm was reported in 75.1%, compared with 29.6% of lesions treated without software. Conversely, a reported margin <5 mm or incomplete ablation was uncommon in software-assisted ablations (0.3% versus 21.4%).

**Table 4:** Achieved ablation margins depending on whether confirmation software was used

Use of confirmation software		Yes	No	*p*-value
Achieved margin sizes	<5 mm or not completed	1 (0.3)	148 (21.4)	<0.001
	5–10 mm	91 (24.7)	294 (42.4)	
	>10 mm	277 (75.1)	205 (29.6)	
	(Missing)	0 (0.0)	46 (6.6)	

For 21 lesions, both use of confirmation software and achieved margin sizes are missing

Because confirmation software use was closely linked to cross-sectional imaging, a post-hoc analysis was performed using a combined guidance/software variable. Compared with CT/MRI-based guidance without confirmation software, CT/MRI-based guidance with software was associated with lower odds of local failure (adjusted OR 0.1, 95% CI 0.0–0.2, *p*<0.01), whereas percutaneous US-only guidance was associated with higher odds of local failure (adjusted OR 1.8, 95% CI 1.1–2.8, *p*=0.02; Supplementary Table 5). This exploratory analysis compared procedural workflows and did not estimate an independent effect of confirmation software.

### Time-to-Event Outcomes According to Confirmation Software use

**Fig. 1:**
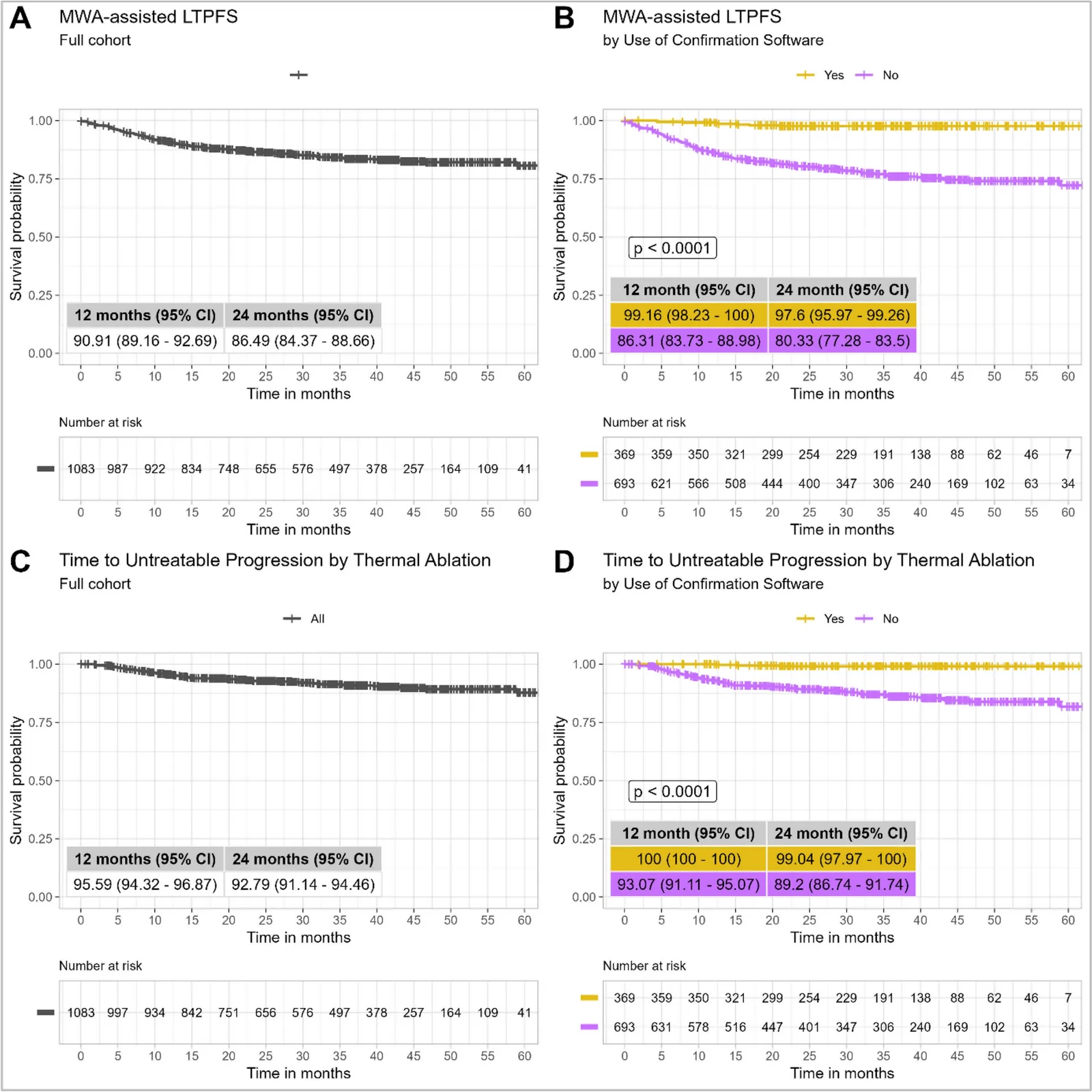
MWA-assisted Local Tumour Progression-Free Survival (LTPFS) and time to untreatable progression by thermal ablation for the full cohort and depending on the use of confirmation software. Kaplan Meier curves and estimates for 12 months and 24 months rates and the respective 95% CI are shown for the full cohort (**A**, **C**) and stratified by the use of confirmation software (**B**, **D**)

MWA-assisted LTPFS was used to describe lesion control from the first MWA to residual disease or recurrence of a target lesion that was not successfully retreated with MWA. For the entire cohort, MWA-assisted LTPFS was 90.9% (95% CI 89.2–92.7) at 12 months and 86.5% (95% CI 84.4– 88.7) at 24 months (Fig. 1A). When stratified by confirmation software use, lesions for which margins were confirmed with software showed markedly higher MWA-assisted LTPFS compared with those treated without software-based confirmation (Fig. 1B; log-rank *p *< 0.0001). The 12 month MWA-assisted LTPFS in the group using confirmation software was 99.2% (95% CI 98.2–100.0) versus 86.3% (95% CI 83.8–89.0) without software. This pattern was consistent across lesion-size subgroups (< 10 mm, 10–20 mm, and >20 mm; Supplementary Fig. 1). The 12 month TTUP by ablation rate was 95.6% (95% CI 94.3–96.9) for the whole cohort (Fig. 1C), 100% (95% CI 100–100) for lesions where confirmation software was used, and 93.1% (95% CI 91.1–95.1) for lesions treated without confirmation software (Fig. 1D).

### Centre-Level Variation in Local Tumour Control

**Fig. 2:**
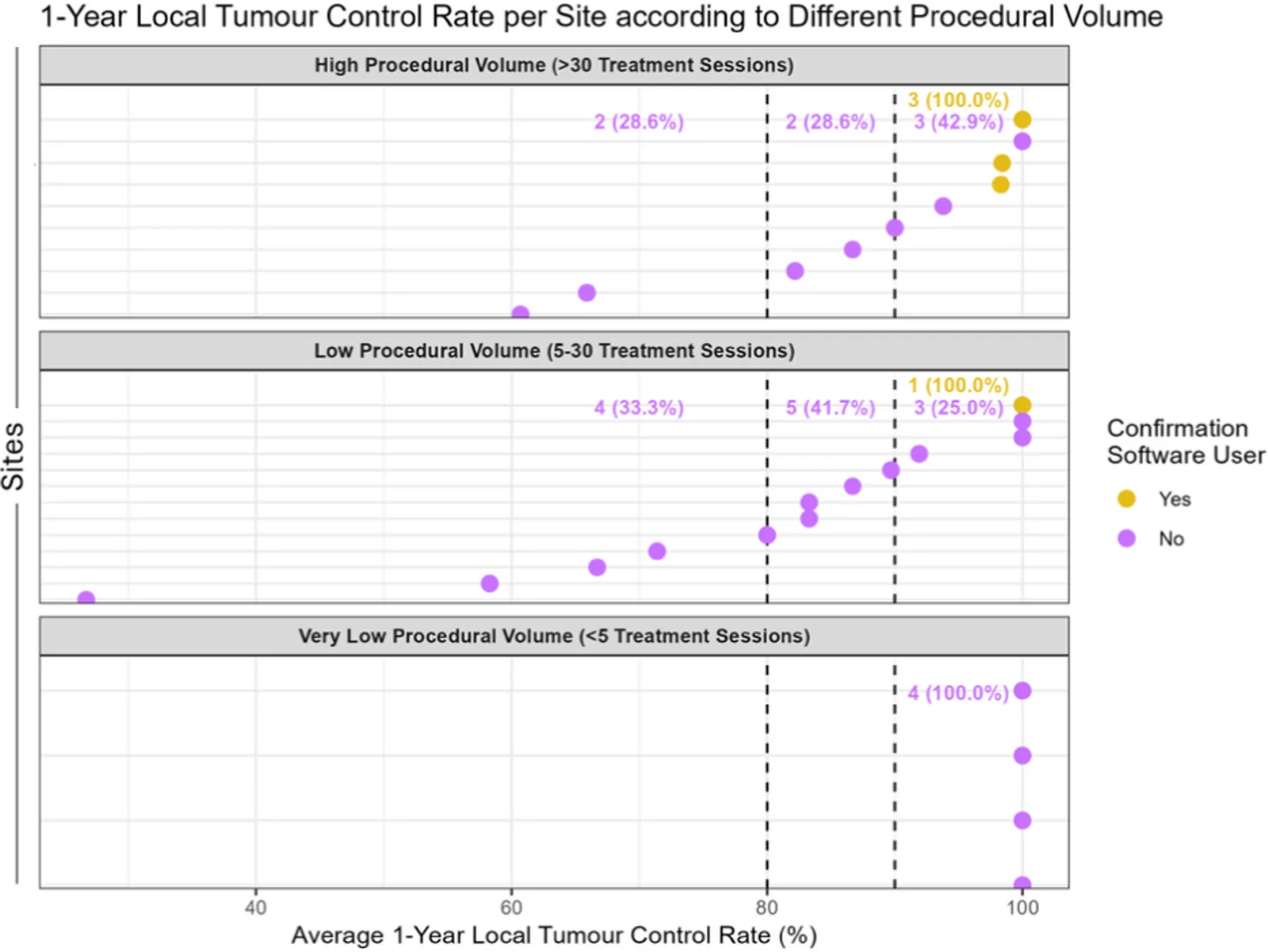
1-Year local tumour control rate per site according to procedural volume category over the course of the study, stratified by confirmation software use. Each dot represents one site (coloured dot). Within each procedural volume category, sites are ordered by increasing local tumour control rate. Dashed vertical lines indicate the 80% and 90% local tumour control rate thresholds. Summary labels indicate the number and percentage of sites in each confirmation software group falling within the corresponding local tumour control rate range: <80%, 80 – <90%, or ≥90%. Procedural volume was calculated over the full study period and was not normalised for the time since site enrolment

To explore whether centre experience may partly compensate for the absence of confirmation software, local tumour control rates were summarised by centre according to procedural volume category and confirmation software use (Fig. 2). Among centres with high procedural volume (> 30 treatment sessions), non-software users showed heterogeneous outcomes: 3 of 7 centres (42.9%) achieved local tumour control rates ≥90%. In centres with low procedural volume (5–30 treatment sessions), non-software users more frequently had local tumour control rates below 90%, with only 3 of 12 centres (25.0%) achieving ≥90%. In contrast, all centres using confirmation software achieved local tumour control rates ≥90%, although only one of those performed <30 treatment sessions.

## Discussion

In this large multicentre real-world study, MWA achieved high local tumour control, with a 1 year per-lesion local tumour control rate of 88.9% and a 12 month MWA-assisted LTPFS of 90.9% when using MWA to control recurrence. These outcomes, obtained across diverse institutions, operators, and procedural workflows, provide insight into the use of MWA in routine clinical practice. Building on available literature from more standardised trial settings [7, 16], and from less robust retrospective series [6, 10, 11, 17, 18], these results underscore the effectiveness of MWA for selected CRLM patients treated with curative intent. Importantly, ablation margins of 5–10 mm and >10 mm were independently associated with lower odds of local failure than margins <5 mm, and a supplementary direct comparison showed lower odds of local failure for margins >10 mm than for margins of 5–10 mm. Exploratory post hoc analyses showed that confirmation software use was associated with wider reported margins and higher MWA-assisted LTPFS.

Margins >10 mm may provide additional local-control benefit beyond margins of 5–10 mm. However, the present observational analysis did not evaluate whether pursuing wider margins increased the risk of injury to adjacent vascular or biliary structures, loss of functional liver parenchyma, or other procedural complications. Therefore, margins >10 mm should not be interpreted as a universal procedural target. Rather, operators should aim for the widest safely achievable complete margin, balancing local tumour control against anatomical constraints and procedural safety. Confirmation software may be particularly valuable in supporting this patient- and lesion-specific balance rather than simply maximising ablation volume.

Previous studies have already shown the value of confirmation software, including the randomised phase 2 COVER-ALL trial [9, 12–14, 19] and﻿﻿﻿ standardising its use is a key goal of ongoing research (ACCLAIM trial; NCT05265169 and New Comet; NCT05129787). This also aligns with the recommendations from a recent international multisociety Delphi consensus paper [20]. The benefit of confirmation software in our study was reflected in wider reported margins and the markedly higher MWA-assisted LTPFS observed in the software group (Fig. 1B), supporting prospective evaluation of confirmation software as a potential component of high-quality ablation workflows [12–14, 21]. Our data showed 99.2% (95% CI 98.2 – 100.0) MWA-assisted LTPFS at 12 months for lesions treated with confirmation software compared with 86.3% (83.8–89.0) for lesions treated without confirmation software (*p* < 0.0001). Although endpoint definitions differed between studies, our 12 month MWA-assisted LTPFS are broadly in line with previously reported local outcomes from the COLLISION trial [7] and NOLA registry [18]. In COLLISION, 12 month lesion-based LTPFS similarly exceeded 90%, while the NOLA registry reported local tumour progression at 12 months in 10.0% of lesions (95% CI: 7.4%-13.0%).

However, in routine clinical practice confirmation software is not widely adopted, as reflected by the fact that 64% of lesions were treated without confirmation software. At the same time, our data also show that adequate margins can be achieved without confirmation software, as 72% of lesions treated without confirmation software had margins ≥5 mm (Table 4). In the lesion-size subgroup analysis, 12 month MWA-assisted LTPFS without confirmation software remained high for lesions <10 mm but was lower for lesions measuring 10–20 mm and >20 mm, supporting a greater potential benefit of software guidance as lesion size increases (Supplementary Fig. 1). Furthermore, stratification by centre experience revealed that, despite considerable heterogeneity across sites, certain centres consistently achieved high local tumour control rates without the use of confirmation software (Fig. 2). Therefore, ablation should adhere to procedural and practice standards, as proposed in a recent consensus paper [22].

Beyond the technical aspects of margin achievement and complementary software platforms, these findings fit within the broader shift in the multidisciplinary management of CRLM, where thermal ablation is increasingly recognised as a definitive local curative therapy rather than a salvage procedure [8, 23, 24]. Meta-analyses have shown that, when selection bias is minimised, survival after ablation approaches that of surgery [5]. More recently, the pivotal COLLISION phase III trial further demonstrated a high likelihood of non-inferiority of thermal ablation compared with surgical resection for ≤ 3 cm deemed resectable or ablatable CRLM, with a substantially lower complication burden. The present results add to these findings by showing that, with intra-procedural confirmation tools, percutaneous MWA in a real-world setting achieves 1-year local tumour control rates equivalent to surgical outcomes, but with lower morbidity [25]. This also aligns with the AmCORE registry, which documented a steady improvement in ablation outcomes over the last decade, largely attributable to technological refinement and improved image guidance [6].

Lastly, in our analysis, percutaneous US guidance without cross-sectional imaging was associated with worse local tumour control compared with CT/MRI-based guidance, which was not the case for US guidance without cross-sectional imaging used during open or laparoscopic procedures. In a recent single-centre cohort of percutaneous US-guided CRLM ablation, intraprocedural CT control was associated with substantially lower local tumour progression than US-guided ablation without CT control, while US-only treatment appeared acceptable mainly for very small, clearly visualised lesions [26]. This is consistent with the broader concept that local efficacy after CRLM ablation is strongly margin dependent. Quantitative margin assessment may therefore be particularly relevant when conventional US provides limited tumour or ablation-zone visibility, a limitation that fusion imaging may mitigate, as CT/US fusion has been shown to improve the visibility and targeting of liver metastases that are poorly visualised on conventional US [27].

Limitations of this study are related to its observational single-arm design and use of extensive remote monitoring and automated data checks instead of source data verification. Confirmation software use was not randomised, and formal adjustment for centre-level clustering was limited by sparse local failures and limited within-centre variation in software use. Residual confounding by operator experience, institutional expertise, imaging resources and case selection therefore cannot be excluded. Additionally, centre experience could not be formally assessed, as it had not been defined a priori and CIEMAR did not capture broader centre-level experience beyond study-specific data. Margin assessment was not standardised across participating centres and the date recorded in the eCRF did not reliably distinguish the initial procedural assessment from subsequent image review or documentation.

## Conclusion

In this multicentre real-world cohort, microwave ablation of colorectal liver metastases achieved a high 12 month local tumour control rate, particularly when adequate margins were achieved. Larger margins were independently associated with a lower risk of local failure. Exploratory analyses showed that confirmation software use was associated with wider reported margins and higher MWA-assisted LTPFS, warranting prospective evaluation. Results confirm that microwave ablation is an effective and safe treatment for selected patients with colorectal liver metastases.

## Supplementary Information

Below is the link to the electronic supplementary material.

Supplementary file1 (DOCX 2285 KB)
