# Supplementary material for: Results from the Pan-European CIEMAR study: Local Tumour Control After Microwave Ablation of Colorectal Liver Metastases and Exploratory Associations with Ablation Margins and Confirmation Software
Source: Cardiovasc Intervent Radiol. 2026 Sep 6;49(10):2004–17. doi: 10.1007/s00270-026-04578-1 (PMC13642442; doi:10.1007/s00270-026-04578-1)
Supplement: Supplementary file 1 [file 270_2026_4578_MOESM1_ESM.docx]

Supplementary Table 1: Treatment sessions details

| **Treatment sessions n = 679** | | **n (%) or value** | |
| --- | --- | --- | --- |
| **Simultaneous resection performed** | | | |
|  | No | | 615 (90.6) |
|  | Yes | | 64 (9.4) |
| **Anaesthesia** | | | |
|  | General anaesthesia | | 361 (53.2) |
|  | Deep sedation | | 125 (18.4) |
|  | Analgesia | | 26 (3.8) |
|  | Neuroleptanalgesia | | 167 (24.6) |
| **Prophylactic antibiotics given** | | | |
|  | No | | 311 (45.8) |
|  | Yes | | 368 (54.2) |
| **Treatment technically successful** | | | |
|  | Yes | | 677 (99.7) |
|  | No | | 2 (0.3) |
| **Hospital stays in nights (categorised)** | | | |
|  | Missing | | 2 (0.3) |
|  | 0-1 | | 446 (65.7) |
|  | 2-4 | | 180 (26.5) |
|  | ≥5 | | 51 (7.5) |

Supplementary Table 2: Adverse Events

|  |  | **Participants n (%)** | **Total AEs** |
| --- | --- | --- | --- |
| **Any AE of any relationship** | | | |
|  | Grade 1 | 52 (10.6%) | 71 |
|  | Grade 2 | 48 (9.8%) | 60 |
|  | Grade 3 | 55 (11.2%) | 83 |
|  | Grade 4 | 17 (3.5%) | 22 |
|  | Grade 5 | 8 (1.6%) | 11 |
| **Related or likely related to the procedure** | | | |
|  | n | 80 (16.3%) | 121 |
| **Grade 3 or 4 related or likely related to the procedure** | | | |
|  | n | 26 (5.3%) | 34 |
| **Grade 5 related or likely related to the procedure** | | | |
|  | n | 0 (0%) | 0 |

Supplementary table 3: Tumour control rate sensitivity analysis

| **Assumption** | | **Controlled lesions/total lesions** | **12-month local control** |
| --- | --- | --- | --- |
| **Primary (complete case)** | | | |
|  | Only lesions with observed 12-month imaging included | 842/947 | 88.9% |
| **Best-case scenario** | | | |
|  | All 136 without 12-month data are assumed controlled | 978/1083 | 90.3% |
| **Worst-case scenario** | | | |
|  | All 136 lesions without 12-month data are assumed not controlled | 842/1083 | 77.7% |
| **Competing-risk scenario (death with disease progression <12 months = failure; 40 lesions)** | | | |
|  | Lesions from patients who died due to disease progression within 12 months were classified as failures; all other lesions without evaluable 12-month outcomes remained excluded. | 842/987 | 85.3% |

Supplementary Table 4: Predictors of 1-year local tumour control using a 5–10 mm ablation margin as reference

|  | **Variable** | **Controlled** | **Not controlled** | **OR (univariable)** | **OR (multivariable)** |
| --- | --- | --- | --- | --- | --- |
| **ECOG status** | | | | | |
|  | 0 | 600 (76.8) | 69 (67.6) | - | - |
|  | ≥1 | 181 (23.2) | 33 (32.4) | 1.6 (1.0-2.5, p=0.04) | 2.5 (1.5-4.3, p<0.01) |
| **Guidance method^1^** | | | | | |
|  | CT or MRI +/- US or other | 591 (70.2) | 55 (52.4) | - | - |
|  | US or open palpation (laparoscopic/surgery) | 115 (13.7) | 7 (6.7) | 0.7 (0.3-1.4, p=0.31) | 0.9 (0.3-2.0, p=0.75) |
|  | US (percutaneous) | 134 (15.9) | 43 (41.0) | 3.5 (2.2-5.4, p<0.01) | 3.6 (2.2-5.9, p<0.01) |
| **Lesion Size** | | | | | |
|  | ≤20 mm | 654 (77.8) | 59 (56.2) | - | - |
|  | >20 mm | 187 (22.2) | 46 (43.8) | 2.7 (1.8-4.1, p<0.01) | 1.7 (1.0-2.7, p=0.03) |
| **Margins Size** | | | | | |
|  | 5-10 mm | 307 (38.5) | 41 (40.2) | - | - |
|  | <5 mm | 84 (10.5) | 42 (41.2) | 3.7 (2.3-6.1, p<0.01) | 3.2 (1.9-5.5, p<0.01) |
|  | >10 mm | 406 (50.9) | 19 (18.6) | 0.4 (0.2-0.6, p<0.01) | 0.3 (0.2-0.6, p<0.01) |
| *Number in dataframe = 947, Number in model = 841, Missing = 106, AIC = 518, C-statistic = 0.798, H&L = Chi-sq(8) 8.41 (p=0.395)* | | | | | |

Supplementary Table 5: Sensitivity analysis of factors associated with loss of 1-year local tumour control, incorporating guidance method and confirmation software use

|  | **Variable** | **Controlled** | **Not controlled** | **OR (univariable)** | **OR (multivariable)** |
| --- | --- | --- | --- | --- | --- |
| **ECOG status** | | | | | |
|  | 0 | 600 (76.8) | 69 (67.6) | - | - |
|  | ≥1 | 181 (23.2) | 33 (32.4) | 1.6 (1.0-2.5, p=0.04) | 2.9 (1.7-4.9, p<0.01) |
| **Guidance method^1^** | | | | | |
|  | CT or MRI +/- US or other no confirmation software used | 276 (32.9) | 50 (47.6) | - | - |
|  | CT or MRI +/- US or other with confirmation software used | 312 (37.2) | 5 (4.8) | 0.1 (0.0-0.2, p<0.01) | 0.1 (0.0-0.2, p<0.01) |
|  | US or open palpation (laparoscopic/surgery) | 115 (13.7) | 7 (6.7) | 0.3 (0.1-0.7, p=0.01) | 0.3 (0.1-0.6, p<0.01) |
|  | US (percutaneous) | 134 (15.9) | 43 (41.0) | 1.8 (1.1-2.8, p=0.02) | 1.8 (1.1-2.8, p=0.02) |
| **Lesion Size** | | | | | |
|  | ≤20 mm | 654 (77.8) | 59 (56.2) | - | - |
|  | >20 mm | 187 (22.2) | 46 (43.8) | 2.7 (1.8-4.1, p<0.01) | 1.8 (1.1-2.8, p=0.01) |
| *Number in dataframe = 947, Number in model = 878, Missing = 69, AIC = 544.3, C-statistic = 0.777, H&L = Chi-sq(8) 5.78 (p=0.672)* | | | | | |

Supplementary Figure 1: **MWA-assisted Local tumour progression-free survival (LTPFS) stratified by tumour size and confirmation software use.** Kaplan–Meier curves showing MWA-assisted LTPFS after microwave ablation according to baseline lesion size. Panels A, C, and E show lesion progression-free survival in the full cohort for lesions measuring <10 mm, 10–20 mm, and >20 mm, respectively. Panels B, D, and F show MWA-assisted LTPFS for the same lesion-size categories stratified by use of confirmation software. Yellow lines indicate lesions treated with confirmation software and purple lines indicate lesions treated without confirmation software. Estimated MWA-assisted LTPFS rates at 12 and 24 months with 95% confidence intervals are shown within each panel. Numbers at risk are displayed below each plot. P-values in stratified panels are from log-rank tests comparing lesions treated with versus without confirmation software.
